# CT and MRI in Urinary Tract Infections: A Spectrum of Different Imaging Findings

**DOI:** 10.3390/medicina57010032

**Published:** 2021-01-01

**Authors:** Mohamed Abou El-Ghar, Hashim Farg, Doaa Elsayed Sharaf, Tarek El-Diasty

**Affiliations:** Radiology Department, Urology and Nephrology Center, Mansoura University, 35516 Mansoura, Egypt; maboelghar@yahoo.com (M.A.E.-G.); dr_doaa383@yahoo.com (D.E.S.); teldiasty@hotmail.com (T.E.-D.)

**Keywords:** urinary tract, infection, pyelonephritis, cystitis, CT, MRI

## Abstract

There are many acute and chronic infections affecting the urinary tract including bacterial, fungal and viral infections. Urinary tract infections (UTIs) can present in many different patterns with variable degrees of severity varying from asymptomatic and uncomplicated forms to life threatening complicated infections. Cross-sectional imaging techniques—including both computed tomography (CT) and magnetic resonance imaging (MRI)—have become very important tools not only for evaluation of UTIs, but also for detection of associated complications. Selection of either CT or MRI in the UTI evaluation depends on several factors such as the presence of contraindication, experience, cost and availability. CT and MRI help in early detection and management of UTIs that reduce the prevalence and severity of complications. In this article we will present the radiologic findings at CT and MRI in different types of upper and lower UTIs including acute pyelonephritis, intrarenal and perinephric abscesses, pyonephrosis, chronic pyelonephritis, emphysematous UTIs, xanthogranulomatous pyelonephritis, tuberculosis (TB), bilharziasis, fungal infection, corynebacterium infection, ureteritis, cystitis, prostatitis, prostatic abscess and urethritis.

## 1. Introduction

Worldwide, urinary tract infection (UTI) is considered the commonest urologic problem, affecting about 150 million patients annually [1]. UTIs account for the greatest proportion (31%) of nosocomial infections in American medical intensive care units while it represents the second most common nosocomial infection after respiratory infection in Europe [2]. The anatomic structure of the urinary tract increases its susceptibility for colonization by several microbial agents [3,4]. UTIs can be caused by many types of micro-organisms, including Gram-positive bacteria, Gram-negative bacteria and certain fungi. Uropathogenic *Escherichia coli* (UPEC) is the commonest causative organism for both complicated and uncomplicated UTIs [5,6,7,8]. Undetected and untreated acute pyelonephritis may lead to renal hypertension, renal scarring, and even renal failure [9].

Diagnostic imaging for evaluation of UTI is mandatory in the following conditions: (a) uncertainty of symptoms; (b) assessment of the complicated UTI causes such as tumors and strictures; (c) persistent and recurrent symptoms despite adequate treatment; (d) assessment of complications that need urgent intervention and management, such as renal abscess [10].

Cross-sectional imaging including both computed tomography (CT) and magnetic resonance imaging (MRI) becomes a part of a routine study of patients with symptomatic and complicated UTIs.

Both MRI and CT provide high image quality, allowing easy diagnosis of urinary tract infections. CT is usually readily available than MRI [11], however both modalities allow comprehensive evaluation of the renal parenchyma, surrounding structures and spaces, and better evaluation of the urothelial wall [12,13].

CT is the ideal imaging modality in the assessment of most cases of UTI as it is a fast technique and provides adequate anatomical and physiological details, identifies both renal and extra-renal pathologies, and provides different phases to be evaluated after intravenous contrast injection. Other CT advantages include the use of multiplanar reconstruction (MPR), curved planar reformatted images, maximum intensity projection (MIP) and three-dimensional (3D) reconstruction. The detectability of air and calcifications is usually better using CT scan than MRI, although these conditions can also be detected on MRI [2,11,13,14,15].

MRI is currently considered as a problem-solving imaging technique if CT is not diagnostic. MRI is recommended also when CT cannot be performed such as in pregnant women and in patients with contrast medium contraindication. Additionally, MRI can be used for diagnosing chronic UTI with or without using contrast material. MRI has also the advantage of high soft-tissue resolution and no radiation exposure [11,16].

Better tissue characterization can be obtained using dynamic contrast enhanced MRI and diffusion weighted MRI [16,17]. Diffusion-weighted imaging (DWI) can easily diagnose pyelonephritis based on renal parenchymal apparent diffusion coefficient (ADC) values, which are usually found to be lower than those of the unaffected renal parenchyma [11,18,19].

MRI is ideal for detection of complications related to UTIs such as renal abscess and also to follow up the response to medical treatment and percutaneous drainage, especially in young patients. Sometimes, delayed excretory phase imaging (either with CT or MRI) is required to evaluate the urinary collecting system [16,20].

CT carries the risk of radiation exposure and side effects of iodinated contrast medium [21]. On the other hand, MRI is not routinely used in the evaluation of UTIs and is reserved for problematic cases due to high cost, limited availability, and scanning duration [22].

In this review, we will explore the respective role of CT and MRI in the evaluation of UTIs. The role of MRI will be highlighted in the diagnosis of UTIs and associated complications, especially when there is a contraindication to CT.

## 2. Cross-Sectional Imaging Features of Urinary Tract Infections

### 2.1. Acute Pyelonephritis

Acute pyelonephritis (APN) is an acute infection of the renal parenchyma and collecting system. Pathophysiology of APN explains its symptoms. It usually results from either ascending infection from the bladder or by hematogenous spread that is why the patients presented with high-grade fever, flank or suprapubic pain, dysuria, urinary frequency, nausea, or vomiting [2]. APN usually responds to adequate antibiotic treatment [23].

**Figure 1:**
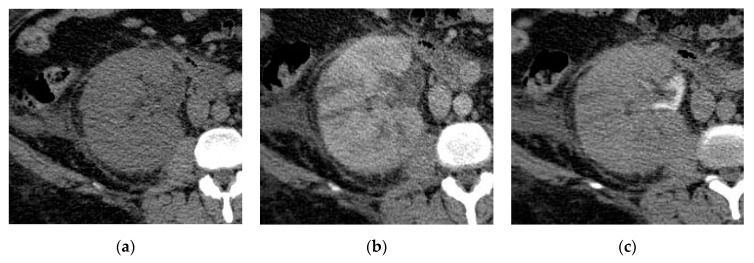
Acute right pyelonephritis. (**a**) Axial non-contrast computed tomography (CT) image shows enlarged right kidney with an irregular outline, thickened perinephric fascia, and distorted perinephric fat. Axial (**b**) early and (**c**) delayed post-contrast CT images show linear streaks extending from the papillae to the cortex with an impaired enhancement of the parenchyma.

A non-contrast CT scan is important for detecting air bubbles in the urinary tract, hydronephrotic changes, and stones. High attenuation areas on non-contrast CT represent hemorrhage, while low attenuation areas represent edema. In early contrast-enhanced CT scan, there is a loss of corticomedullary differentiation associated with wedge-shaped hypo-enhancing areas or striated nephrogram pattern of linear alternating streaks of increased and decreased attenuation extending from the renal sinus to the renal cortex (Figure 1) [11]. The pathophysiology of these radiological features may be due to poorly functioning renal parenchyma caused by vasospasm, tubular obstruction, or interstitial edema [10,24]. Additionally, perinephric fat stranding and renal abscess formation may be noted at CT imaging [23].

Some radiological features may suggest specific pathologies, for example, the thick enhancing wall of the renal pelvis without renal parenchymal affection indicates pyelitis [25].

**Figure 2:**
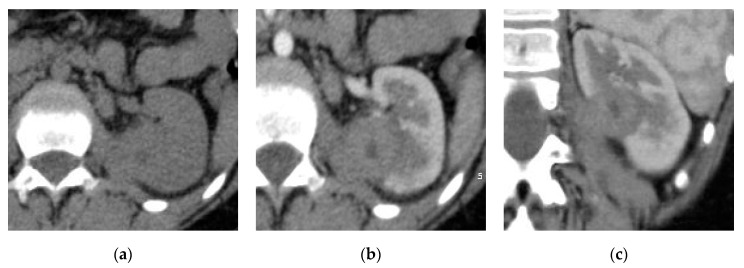
Left hematogenous acute pyelonephritis. (**a**) Axial non-contrast CT image shows focal contour bulge at the posterior parapelvic region of the left kidney. (**b**) Axial and (**c**) coronal reformatted post-contrast CT images show mildly enhancing rounded lesion at the mid-zone of the kidney with adhesion to the left psoas muscle mimicking renal tumor.

**Figure 3:**
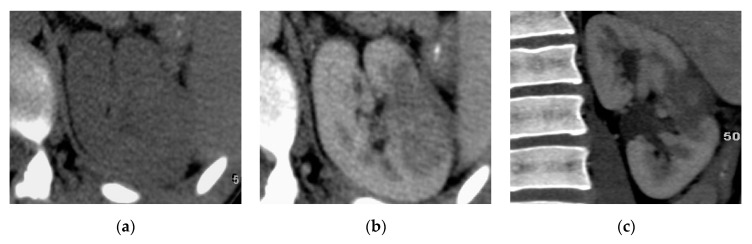
Left acute pyelonephritis. (**a**) Axial non-contrast CT image shows a slight focal contour bulge at the middle of the kidney. (**b**) Axial and (**c**) coronal reformatted post-contrast CT images show mildly enhancing subcapsular lesion of relative wedge shape extending to the medulla with thickened related perinephric fascia with distorted perinephric fat.

The presence of round peripheral hypo-enhancing parenchymal renal lesions with a clinical presentation of pyelonephritis favors the diagnosis of the hematogenous route of infection (Figure 2), however the ascending infection usually presents with wedge-shaped areas (Figure 3). When the entire kidney is involved the differentiation between the two routes is difficult [24].

CT is also important for differentiating pyelonephritis from tumor and renal infarction. In certain cases, if the diagnosis is not established, a delayed CT scan after three hours should be performed to assess retention of contrast that differentiates pyelonephritis from renal tumors and renal infarctions. Imaging features of acute pyelonephritis usually show significant improvement after appropriate medical treatment but the tumor will not. Sometimes, radiological signs of APN lag behind clinical improvement and this can create clinical uncertainty and require histopathological confirmation. Extrarenal manifestations of APN that are observed on CT include periportal edema, gall bladder wall thickening, and renal vein or inferior vena cava thrombosis [2], but are rarely observed.

MRI signs of acute pyelonephritis are similar to those detected at CT scan; renal enlargement, alterations of signal intensity due to hemorrhage or edema, striated nephrogram, and perinephric fluid reaction. On MRI, signal void lesions detected in the urinary tract may be due to either calculi or air bubbles. With MRI, APN shows a heterogeneous lesion of hypointense signal intensity on T1-WI and hyperintense signal intensity on T2-WI. MRI provides high soft-tissue contrast resolution, facilitating differentiation of APN from renal scarring, and detection of the extrarenal extension [1,2].

MRI with contrast helps to depict areas of renal parenchyma involvement. Gadolinium-enhanced inversion recovery imaging can help to detect APN accurately in children compared with dimercaptosuccinic acid (DMSA) scintigraphy. This method accentuates contrast differences as unaffected renal tissue enhances normally. However, APN remains hypointense after the administration of intravenous contrast media. MR urography (MRU) using heavy T2 weighted sequence can detect hydronephrotic changes in cases of urinary tract obstruction and extension of perirenal inflammation, particularly when fat suppression sequence is used. DWI can characterize lesions without contrast injection or ionizing radiation. On DWI, APN can be more confidently diagnosed when areas of restricted diffusion are demonstrated; these areas are hyperintense on high b-value DWI, and hypointense on ADC map which accurately differentiate between unaffected renal parenchyma, abscesses and nephritis. DW-MRI demonstrated very high accuracy and diagnostic agreement (~95%) with gadolinium-enhanced MRI [2,26,27,28].

There are two types of pyelonephritis; diffuse type and focal type. The focal type is limited to a mass-like or localized peripheral wedge-shaped hypo-enhancing lesion associated with adjacent fat stranding. In the diffuse form of pyelonephritis, kidney enlargement, poor parenchymal enhancement (delayed nephrogram), perirenal fat stranding, and thickened fascia are usually noted [14].

### 2.2. Renal and Perinephric Abscesses

An abscess may occur secondary to aggregating lesions of infection in pyelonephritis or superimposed infection of a pre-existing urinoma or hematoma [10]. The risk of abscess formation increases in the presence of obstructive uropathy and immunosuppression conditions. Diabetes is also a major risk factor for complicated UTIs including renal abscess and emphysematous pyelonephritis [29]. The commonest infectious organisms in renal abscesses are *Escherichia coli*, and *Staphylococcus Aureus* [14], while Candida species are more commonly detected in diabetic patients. About 20% of patients with a renal abscess may have a negative urine culture [14,25].

**Figure 4:**
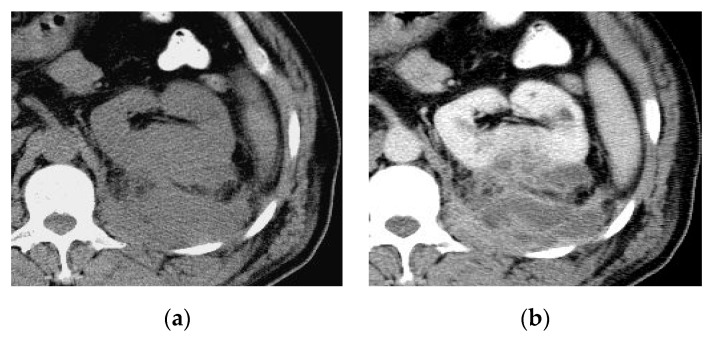
Left renal and perirenal abscess. Axial (**a**) non-contrast and (**b**) post-contrast CT images show multilocular cystic lesion at the posterior aspect of the mid-zone of the left kidney with enhancing septa, it extends to the posterior perirenal space involving the left crus of the diaphragm and posterior abdominal wall.

**Figure 5:**
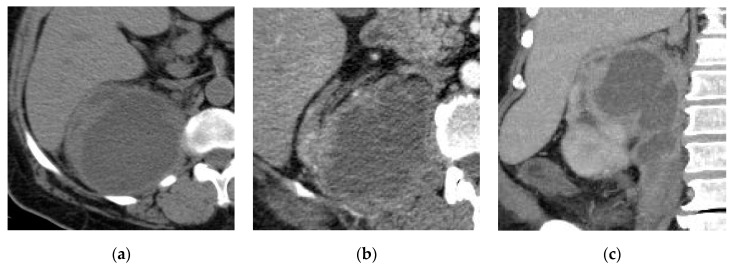
Right renal abscess. (**a**) Axial non-contrast CT image shows right upper polar cystic lesion with a thick wall. (**b**) Axial and (**c**) coronal reformatted post-contrast CT images show upper polar abscess extending to the right psoas muscle with a thick enhancing wall.

**Figure 6:**
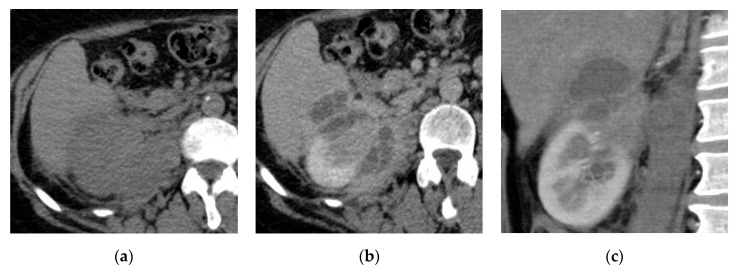
Right renal abscess with hepatic extension. (**a**) Axial non-contrast CT image shows right upper polar isodense lesion with irregular renal contour. (**b**) Axial and (**c**) coronal reformatted post-contrast CT images show multilocular abscess extending to the perirenal space and the liver.

**Figure 7:**
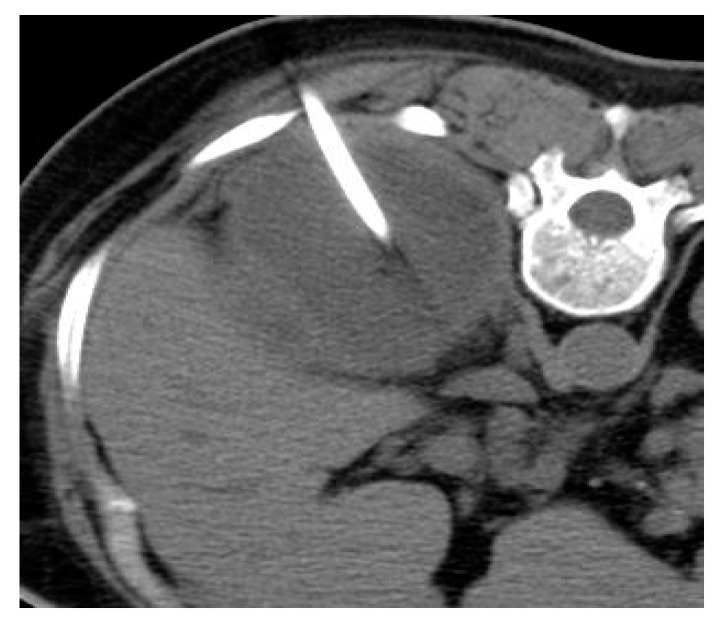
CT guided tube drain of the right renal abscess of the case number 5.

The renal abscess appears as hypodense geographic or rounded non-enhancing fluid collection at contrast-enhanced CT. However, the fluid contents may have high attenuation than the clear fluid in early stages. The enhancing margin of abscess represents a pseudocapsule with variable degrees of wall thicknesses and nodularity. A halo of reduced enhancement may surround the abscess during the nephrographic phase. Sometimes, air bubbles may be seen within the abscess and appear as locules of low density at non-contrast CT scan. The renal fascia is often thickened with soft-tissue stranding; septal thickening and perinephric fat obliteration (Figure 4). CT can easily detect extrarenal extension of the abscess into the perinephric spaces, psoas muscle, and the adjacent organs (Figure 5 and Figure 6). CT guided aspiration and tube drain is a preferable method for minimally invasive treatment of renal abscess in case of difficult ultrasound approach (Figure 7) [1,2,24,25].

**Figure 8:**
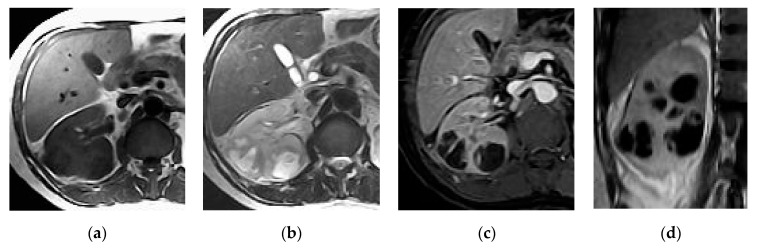
Multiple right renal abscesses. Axial (**a**) T1 and (**b**) T2 weighted images show two cystic lesions at the posterior aspect of the renal lower pole. (**c**) Axial and (**d**) coronal post-contrast GRE T1 weighted images show multiple marginally enhancing abscesses with enhancing septum at the later one at the lower pole.

The appearance of renal abscess at MRI study depends upon the amount of fluid, cellular debris, and protein. So, it appears as a complex cystic lesion with heterogeneous low signal intensity on T1-WI and high signal intensity on T2-WI (Figure 8a,b). The wall of the abscess is usually thick and irregular with a little enhancement on the delayed excretory phase [30]. Renal parenchyma adjacent to the abscess can demonstrate low signal intensity on early phases with subsequently delayed enhancement [15]. Perinephric fat stranding is usually seen adjacent to the renal abscess [31]. Air bubbles can be rarely seen within the cystic lesion and strongly suggests abscess formation. Gadolinium-enhanced MRI can differentiate enhancing lesions from the non-enhancing soft tissue edema (Figure 8c,d).

**Figure 9:**
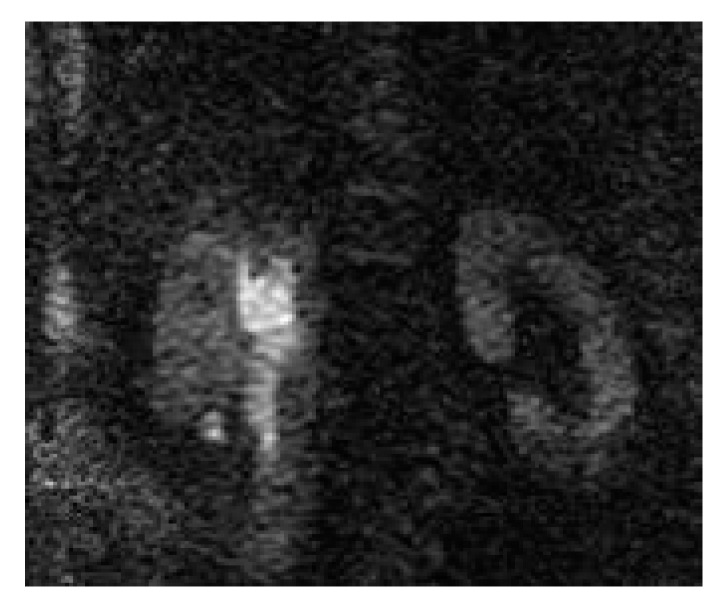
Diffusion-weighted image (DWI) of right renal and perirenal abscess with diffusion restriction.

The fluid contents show characteristically marked and heterogeneous restricted diffusion on DWI due to the presence of viscous pus, which favors the diagnosis of renal abscess rather than renal tumor [30] (Figure 9). DWI also useful in patients’ follow-up with the absence of diffusion restriction indicates a resolution of the infection [2,30].

### 2.3. Pyonephrosis

Pyonephrosis is the collection of pus in an obstructed collecting system; the obstruction may be due to stricture, stone, congenital anomaly, or tumor [14]. Pyonephrosis is an urgent diagnosis and it requires urgent urinary drainage. On CT scan, signs of infection include high urine attenuation and contrast layering in the dilated collecting system, thickening and hyperenhancement of renal pelvis wall, perinephric inflammatory lesions, and renal cortical thinning [1,14]. Contrary to other types of UTIs, CT has limited value in differentiation between pyonephrosis and hydronephrosis [10,24]. Both urinary tract disorders may present with the same radiological signs which may be more prominent in pyonephrosis [25].

**Figure 10:**
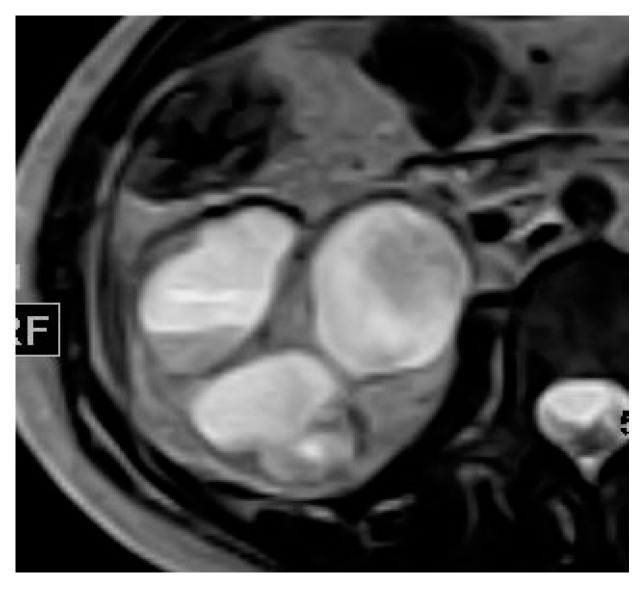
Right pyonephrosis. Axial T2 weighted image shows hydronephrotic changes with fluid–fluid layering inside the dilated calyces.

MRI is preferred over CT in the diagnosis of pyonephrosis as the delineation of obstruction cause and the degree of collecting system dilatation can be obtained using conventional MRI sequences without contrast. At T1-WI, the signal intensity of the infected fluid will be low or slightly high however, it will be intermediate to low at T2-WI. MRI can also detect the fluid–fluid layering and the presence of renal pelvis thickening (Figure 10). DWI may have an added role in differentiating pyonephrosis from hydronephrosis as pyonephrosis demonstrates characteristic diffusion restriction. MRI with contrast may show mural enhancement and thickening of the renal pelvis [14].

### 2.4. Chronic Pyelonephritis

**Figure 11:**
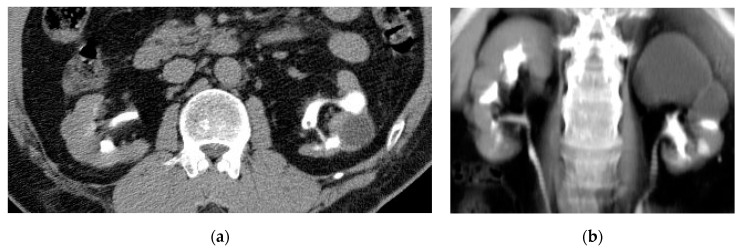
Bilateral chronic pyelonephritis. (**a**) Axial and (**b**) coronal CTU images show small-sized kidneys with irregular contours and localized left upper and middle hydrocalycosis.

**Figure 12:**
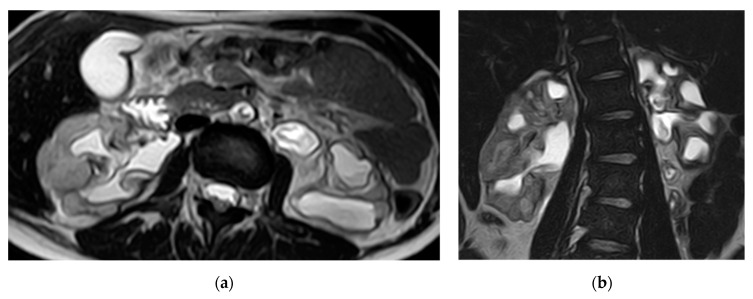
Bilateral chronic pyelonephritis. (**a**) Axial and (**b**) coronal T2 weighted images show renal scarring, and parenchymal atrophy with irregular dilated calyces and irregular contour of both kidneys.

Chronic pyelonephritis may result from recurrent infections or reflux of infected urine in childhood [32]. It is characterized by parenchymal atrophy of the affected areas, hypertrophy of residual normal parenchymal tissue, clubbing of the calyces secondary to retraction of the papilla from adjacent overlying renal scarring, dilatation of the calyces, and overall renal asymmetry. CT with contrast is important to differentiate the non-enhancing areas of infarction from the scar tissue; it also can diagnose the pseudotumors due to focal parenchymal hypertrophy from renal neoplasm, MRI can provide the same information obtained from CT without the need of contrast in most the cases (Figure 11 and Figure 12) [2,25,33]. Lobar infarcts are characterized by their lack of calyceal affection. Persistent fetal lobulations are characterized by parenchymal depressions lying between the calyces rather than opposite the calyces [14].

**Figure 13:**
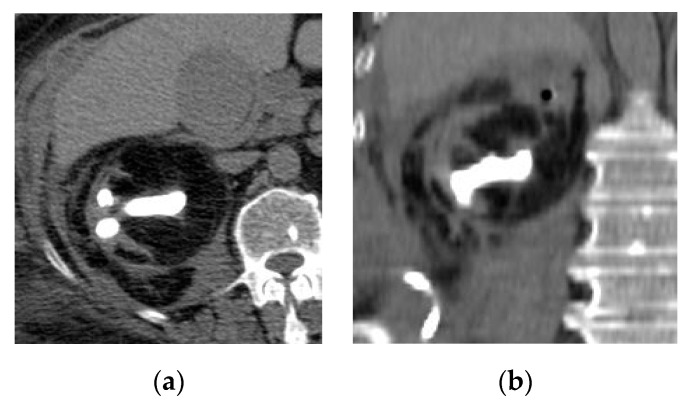
Right renal sinus lipomatosis. (**a**) Axial and (**b**) coronal reformatted non-contrast CT images show small chronic pyelonephritic right kidney with branched hyperdense stone at the renal pelvis and middle calyx. There is a large fatty area of low density replacing the renal sinus and involving the renal parenchyma and the perirenal space.

Renal replacement lipomatosis is seen when there is total atrophy of the renal parenchyma and complete fibrofatty replacement of renal sinus. It is associated with stag horn stone in more than 70% of cases. CT and MRI confirmed the presence of fat in the atrophic kidney and its extension outside the kidney [1]. The calyces are usually stretched and elongated without hydronephrosis (Figure 13).

### 2.5. Xanthogranulomatous Pyelonephritis (XGP)

Xanthogranulomatous pyelonephritis can be considered as an atypical immune response to chronic infected urinary tract obstruction occurring secondary to staghorn calculus. Infectious organisms are commonly *Escherichia coli* and *Proteus mirabilis*. There is no specific symptom of XGP. It usually affects perimenopausal females with a past history of recurrent UTI, urinary obstruction or diabetes [2,4,10,24]. The immune response to chronic infection consists of renal parenchyma replacement by lipid-laden macrophages and chronic destructive granulomatous infection. The condition usually involves the renal pelvis and extends into the renal medulla, cortex, perirenal space, and retroperitoneum. There are two different forms of XGP; a diffuse type and a localized type, the latter being less common [2,3,15,24].

**Figure 14:**
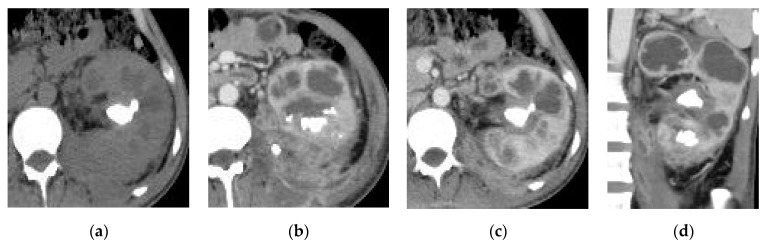
Left xanthogranulomatous pyelonephritis (XGP): (**a**) axial non-contrast CT image shows an enlarged left kidney with hyperdense stone at the renal pelvis and multiple fluid locules at the renal parenchyma. (**b**,**c**) Contrast-enhanced axial and (**d**) coronal reformatted CT images show multiple stones with multiple cavities at the renal parenchyma and irregular inflammatory lesion at the lower pole of the kidney.

**Figure 15:**
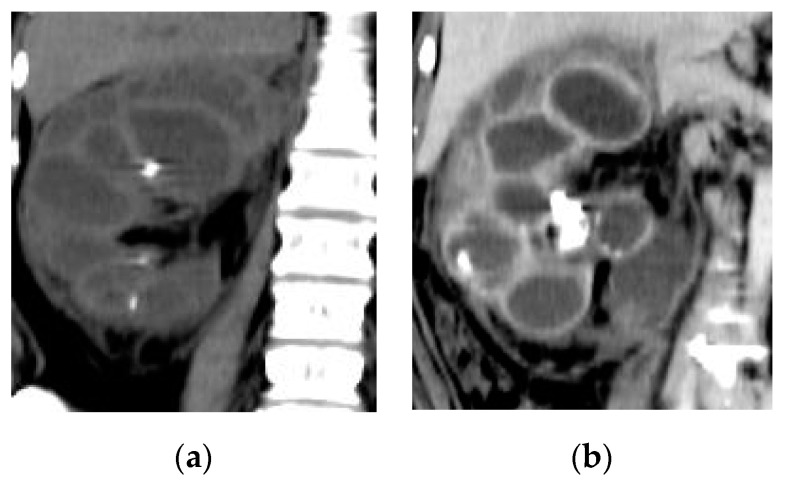
Right XGP. (**a**) Non-contrast and (**b**) post-contrast coronal reformatted CT images show stones at the renal pelvis and middle calyx with multiple parenchymal cavities.

Contrast-enhanced CT is the technique of choice in the assessment of Xanthogranulomatous pyelonephritis; however, MRI can be a useful alternative when iodinated contrast is contraindicated. CT may demonstrate an enlarged kidney replaced with low attenuation dilated calyces resulting in parenchymal atrophy and the classic bear paw sign [4]. These dilated calyces are thought to be filled with debris, hemorrhage, or pus. The combination of a non-functioning enlarged kidney, obstructing stone within non dilated renal pelvis, dilated calyces, and perinephric fat stranding is strongly suggestive of XGP (Figure 14 and Figure 15) [4,24].

Extrarenal XGP extension can be detected with perirenal soft tissue inflammation and abscess formation that extend from the kidney and dissect through the perinephric space, adjacent viscera, and psoas muscle [1].

### 2.6. Emphysematous Urinary Tract Infection

Emphysematous UTI is a life-threatening necrotizing infection seen commonly in diabetic or immunosuppressed patients secondary to infection with gas-forming organisms, such as *Escherichia coli*. Patients with bladder outlet obstruction, neurogenic bladder, and chronic pyelonephritis also represent a high risk group. Emphysematous UTI includes emphysematous pyelonephritis and emphysematous cystitis [25,34]. The clinical presentation is variable, ranging from no symptoms to marked septicemia. Pneumaturia represents the most specific symptom however, it is rarely reported. Early diagnosis of emphysematous UTI is very important, as rapid treatment with intravenous antibiotic therapy is critical to avoid associated morbidity and mortality [35,36,37,38].

**Figure 16:**
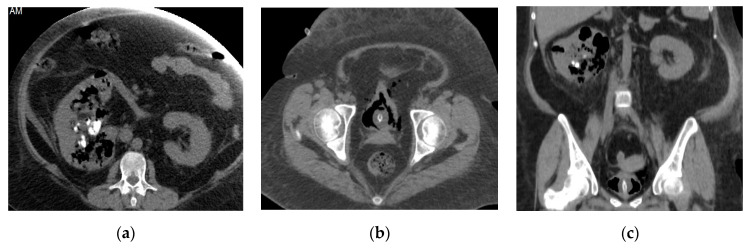
Emphysematous pyelonephritis and cystitis. Axial non-contrast CT images of the abdomen (**a**), pelvis (**b**), and coronal reformatted non-contrast CT image (**c**) show right emphysematous pyelonephritis in the form of diffuse parenchymal enlargement and destruction, multiple bubbly air locules involving the renal parenchyma and extends to the pelvicalyceal system with perirenal inflammatory fat strands and thickened fascia. Emphysematous cystitis can also be detected in the form of intramural air, and air within the bladder lumen with minimal perivesical extension.

Non-contrast CT scan is the key imaging modality for diagnosis of emphysematous UTIs, based on the detection of air bubbles in the renal parenchyma, collecting system, bladder lumen and sometimes in the perirenal and perivesical tissue (Figure 16). CT findings of emphysematous pyelonephritis include diffuse renal enlargement and destruction, linear streaks of air bubbles radiating from the renal papillae, air-fluid layering, and focal tissue liquefaction which may be associated with renal and perirenal abscess. Contrast injection is often contraindicated in patients with renal impairment however, it can be used to evaluate the extent of renal parenchyma destruction. If air locules are only confined to the collecting system, this may suggest emphysematous pyelitis. Sometimes, the isolated distribution of gas in the collecting system may be related to urethral catheterization or uro-intestinal fistula [4,10,24,33,39].

Based on radiological findings, emphysematous pyelonephritis can be classified into two types. Type 1 emphysematous pyelonephritis demonstrates necrosis of renal parenchyma with mottled or streaky areas of air locules without an extrarenal fluid collection. Type 2 emphysematous pyelonephritis demonstrates intrarenal or perirenal fluid collections that are often associated with air locules or air within the collecting system. Type 1 and type 2 mortality rates are reported to be 69% and 18%, respectively [1].

CT-guided drainage followed by intravenous antibiotics can be used for adequate management of emphysematous pyelonephritis particularly in solitary kidney patients, absence of severe toxicity, and in the localized affected kidney part [29].

MRI is not the imaging modality of choice for the diagnosis of emphysematous pyelonephritis, it is used if there is a contraindication to CT. At MRI, the air bubble appears as a signal void on both T1- and T2-WI, but this may mistake with calculi or rapid-flowing blood. Perinephric and intrarenal fluid collections are better delineated on MRI [39].

The classic imaging feature of emphysematous cystitis is the presence of air bubbles in the bladder wall or lumen without a recent history of instrumentation. As mentioned before, CT has high sensitivity in the detection of intramural or intraluminal gas. CT can also evaluate the other causes for intravesical air bubbles, such as a colovesical fistula [37,40]. MRI findings of emphysematous cystitis include inflammatory findings of concurrent cystitis or entero-vesical fistula [36].

### 2.7. Urinary Tuberculosis

The urinary tract is the second most common site affected by tuberculosis after the lungs [41]. In urinary tuberculosis, the urinary bladder is normally involved in a later stage after renal involvement and through the descending route [42]. Symptoms of urinary tuberculosis are nonspecific and include frequency, dysuria, and hematuria. Tuberculosis should be considered in patients with sterile pyuria, and persistent cystitis [41].

The kidneys can be affected in miliary tuberculosis due to the hematogenous spread of Koch’s bacillus. It can produce renal parenchyma ulceration, leading to cavity formation. The spread of bacilli in the urine can also lead to urothelial ulceration, followed by fibrosis and stenosis which are usually located at either the vesicoureteric junction or pelviureteric junction [24]. At a later stage, the reduction in bladder volume, vesical wall calcifications sparing the bladder mucosa, are combined with renal and ureteric calcifications [42].

The earliest radiological finding of TB which can be detected in CT urography (CTU) is dilated calyx with a feathery appearance, which can be seen as a cavity communicating with a deformed calyx or a phantom calyx in later phase [14]. Delayed CTU demonstrates accumulation in the calyces, stenosis of calyceal infundibulum due to fibrosis with proximal ball-shaped hydrocalyx, ureteral stricture and, calcifications of the renal parenchyma with a putty kidney appearance [33].

**Figure 17:**
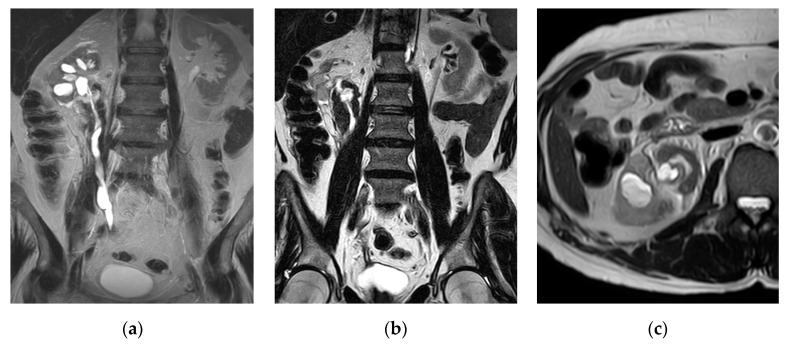
Urinary tuberculosis. (**a**,**b**) Coronal T2-WI and (**c**) axial T2-WI show small size right kidney with chronic pyelonephritic changes in the form of renal scarring and cortical thinning. There is mild hydroureteronephrosis down to the pelvic segment of the ureter with mural thickening of the pelvicalyceal system and ureter associated with clubbing of dilated calyces and dilated ureter with beaded appearance. There is diffuse circumferential periureteric soft tissue mass of low T2 weighted signal intensity surrounding the lumber ureter associated with retroperitoneal fat stranding and thickened fascia.

MRI features of renal macronodular tuberculoma include hypointensity on T1weighted images and a thick, irregular, hypointense peripheral wall with intralesional fluid–fluid layering on T2 weighted images. TB granulomas may appear as mildly enhancing soft-tissue masses on MRI [2]. Ureteric affection may be seen as mural thickening causing strictures, shortening, and beaded appearance. The extra-renal extension can involve periureteric and retroperitoneal tissues (Figure 17) [2,14].

In the acute phase of bladder tuberculosis, CT and MRI features include diffuse irregular bladder wall thickening, irregular mucosal masses due to coalescing tubercles with edema, ulceration, and trabeculation [43]. Vesicoureteric junction orifice may be fixed and patulous, leading to vesicoureteric reflux. In the chronic phase, radiologic findings are a thick-walled contracted bladder due to fibrosis [44]. Associated seminal vesicles calcification also may be detected, but the urinary bladder wall calcification is rare and seen only after healing [43,44,45]. Bladder tuberculosis may be complicated by sinus tract or fistula formation however, these complications are not frequent and can be detected accurately on MRI and CT [41].

### 2.8. Urinary Bilharziasis

Urinary bilharziasis or schistosomiasis is the most common parasitic infection of the urinary tract caused by *Schistosoma haematobium* [42]. It is considered a major health problem in developing countries predisposing patients to squamous cell carcinoma. This parasitic infection generally occurs in the urinary bladder but can spread via reflux to the ureters and kidneys [33,36].

Symptoms of urinary schistosomiasis are usually non-specific and include dysuria, suprapubic pain, and microscopic hematuria. Red and white cells can be detected in urine however, the diagnosis is confirmed only by microscopic detection of eggs in urine [41].

**Figure 18:**
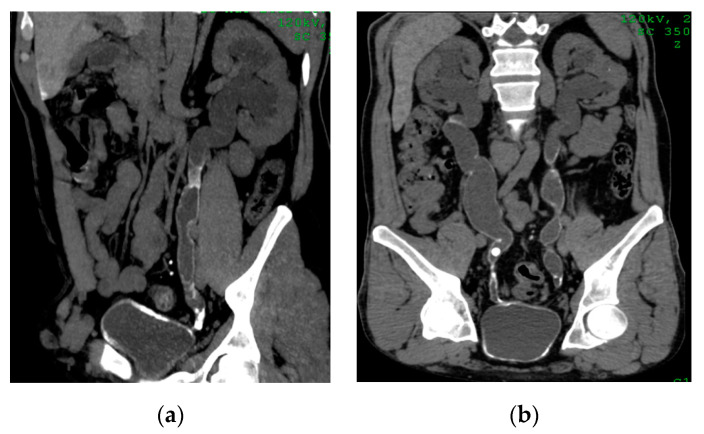
Urinary bilharziasis. (**a**,**b**) Coronal reformatted CT images without contrast showing diffuse mural calcifications of the bladder, and both ureters with bilateral hydroureteronephrosis and multiple bilateral ureteric strictures associated with secondary stone at right pelvic ureter.

Imaging findings mirror the pathologic course. In the acute stage, nodular thickening of the urinary bladder wall is detected easily in MRI. Curvilinear mural calcifications, best visualized on CT scan, in the wall of the bladder or ureter are frequent findings and are mainly caused by calcified dead ova. Typical additional features are strictures of the ureters or reflux [36,42]. The chronic stage is characterized by a contracted, thick calcified bladder wall with ureteric stenosis and calcification (Figure 18). An intravesical mass may also be present secondary to complicating bladder carcinoma, particularly squamous cell carcinoma [24,33,41].

In the majority of patients, the calcifications affect only the bladder mucosa however, in the most severe cases, they reach the muscular layer and the tunica adventitia, leading to fibrosis of the whole urinary bladder, leading to bladder wall deformity. The collection of several granulomas can cause a bilharzioma, taking the appearance of pseudotumor. Lesions are commonly present in the posterior surface of the bladder, the vesical triangle, and around the neck of the bladder. In later stages of urinary bilharziasis, the distal ureters may be involved; renal affection occurs later, secondary to ascending infection [42].

### 2.9. Corynebacterium Infection: Encrusted Pyelitis and Cystitis

**Figure 19:**
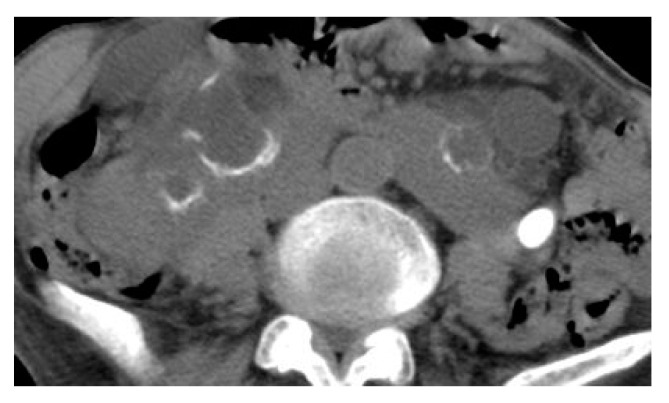
Encrusted pyelitis in horseshoe kidney. Axial non-contrast CT image shows horseshoe kidney with malrotation of both compartments and marginally calcified calyces.

This is a nosocomial type of infection caused by urealyticum bacteria colonizing the urinary tracts of immune-compromised patients. This type of infection usually leads to the formation of ammonium magnesium phosphate calculi which become encrusted in the urothelium of the collecting system and ureter. Non-contrast CT scan is the best imaging technique for visualizing linear hyperdense calcifications along the thickened urothelium (Figure 19). MRI can easily detect mural thickening, perirenal, and periureteric fat stranding [33,46,47].

### 2.10. Urinary Candidiasis:

**Figure 20:**
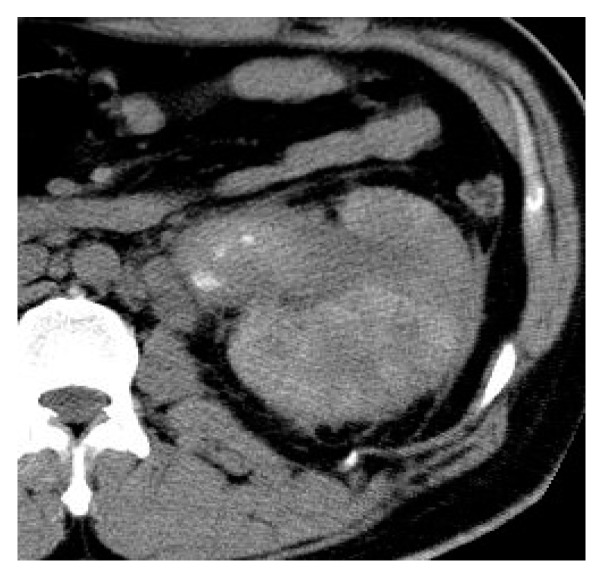
Left renal fungus infection. Axial non-contrast CT image shows hyperdense lesion at the renal pelvis with calcific foci inside.

The urinary tract can be colonized by *Candida albicans* either via hematogenous spread or ascending infection in immune-compromised patients. It may complicate gram-negative UTIs. On contrast-enhanced CT, the mycelial aggregation does not demonstrate enhancement. It has a rolled appearance when it contains gas between the layers of fungal colonies. Calcified lesions indicate healing. Thickening of the renal pelvis and hydronephrosis due to ureteric obstruction by the fungus ball may be seen on CT (Figure 20) [1,33].

At MRI, renal fungal infections usually manifest as parenchymal micro-abscesses demonstrating high signal intensity on T2-WI. Coalescing of small abscesses leads to the formation of a larger parenchymal abscess with or without hydronephrotic changes. Fungus balls are seen as non-enhancing soft tissue mass occupying the collecting system, that often isointense on T1-WI and hyperintense on T2-WI. Fungal infections may lead to local vascular inflammation and thrombosis. Parenchymal calcifications may also occur in chronic fungal infections [48].

### 2.11. Ureteritis

**Figure 21:**
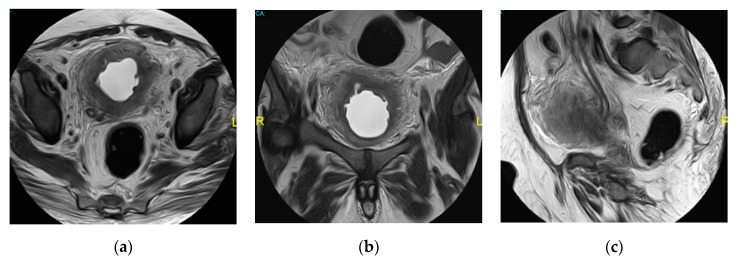
Cystitis and ureteritis. (**a**) Axial and (**b**) coronal T2–W images show diffuse thickened irregular bladder wall, with multiple intramural trabeculation, perivesical fat stranding, and inflammatory reaction. (**c**) Sagittal T2–WI demonstrates diffuse mural thickening of the left ureter with periureteric fat stranding.

Ureteritis refers to ureteric inflammation, which usually occurs as a result of ascending infection secondary to cystitis [49]. Other rare causes include hematogenous spread and chronic infection in a patient with a ureteral stent. CT and MRI demonstrate diffuse mucosal urothelial thickening, often with associated periureteric stranding and inflammatory reaction (Figure 21) [37].

Ureteritis cystica is a very rare benign condition characterized by cystic degeneration of epithelial cell nests invaginating into the lamina propria leading to the formation of multiple small submucosal epithelial-lined cysts. This condition may present with hematuria and is often associated with recurrent or chronic UTI. On CTU or MRU, multiple tiny filling defects are usually seen, and associated hydronephrotic changes may coexist [49].

### 2.12. Cystitis

Cystitis refers to bladder inflammation. It may be isolated or combined with the involvement of the whole urinary tract or adjacent organs [42]. Symptoms include frequency, dysuria, hematuria, urgency, or suprapubic pain. Sources include infection (bacterial, schistosomiasis, TB, parasites, or mycosis), medications (cyclophosphamide) or radiation [2]. Cystitis usually results from ascending urethral infection, such as *Escherichia coli* [37]. It is more common in females due to short urethral length and urethral proximity to the anus [42].

**Figure 22:**
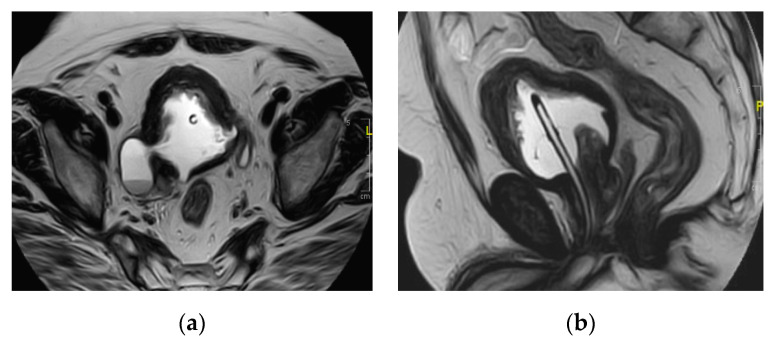
Recurrent cystitis with right vesical wall diverticulum. (**a**) Axial and (**b**) sagittal T2 weighted images of the pelvis demonstrate diffuse irregular bladder wall thickening, and irregular mucosal thickening. Right lateral wall narrow neck diverticulum with fluid–fluid layering inside denoting infected urine. Enlarged prostate with median lobe hypertrophy projecting inside the bladder and Foley’s catheter could be noted.

On CT, radiologic findings of acute uncomplicated bacterial cystitis include either diffuse or focal mural hypertrophy, periserosal edema, and mucosal urothelial irregularity. Layering debris can also be detected and best demonstrated by sonography. Sometimes focal, protruding pseudotumors are reported [34,37,42]. MRI can detect diffuse or focal irregular mural thickening due to edema of the urinary bladder wall. Gadolinium-enhanced MRI of the bladder shows differential enhancement proportional to the severity of inflammation but generally less than that of the tumor [2,36,50]. Radiological evaluation of recurrent cystitis is indicated using CT and MRI, to check for bladder tumor, foreign body, diverticula (Figure 22), or fistulae between the gastrointestinal tract and the bladder [42].

**Figure 23:**
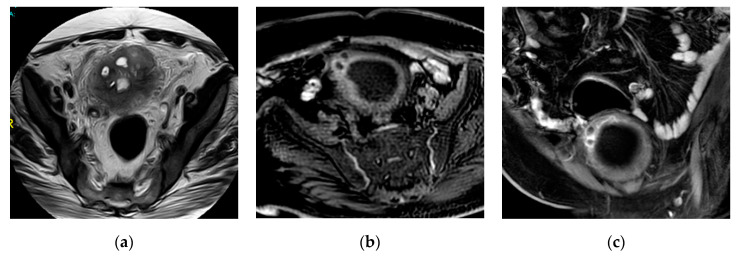
Cystitis complicated by mural bladder abscess. (**a**) Axial T2–WI, (**b**) axial post-contrast T1–WI, and (**c**) coronal post-contrast T1–WI show circumferential thickened irregular bladder wall. The right aspect of the bladder dome shows marked mural thickening with underlying intramural marginally enhancing small fluid locules and distorted related perivesical fat, the radiological features suggest bladder wall abscesses.

**Figure 24:**
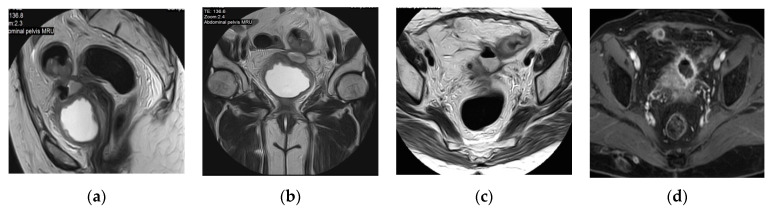
Cystitis complicated by an exophytic bladder wall abscess in a patient with colonic diverticulitis. (**a**) Sagittal, (**b**) coronal, (**c**) axial T2-W images and (**d**) post-contrast axial T1–WI show diffuse irregular bladder wall thickening with contiguous soft tissue inflammatory thickening closely related to adjacent margin of a thick-walled sigmoid colon associated with fluid-like collection demonstrating irregular peripheral enhancement along the superior aspect of the urinary bladder. Diffusely thickened adjacent intestinal loops with multiple diverticulitis showing peridiverticular fat stranding and mural enhancement could be noted.

Sometimes, acute infectious cystitis may be complicated by a mural bladder abscess formation, which can be demonstrated by MRI and CT as an intramural (Figure 23) or exophytic (Figure 24) non-enhancing cystic collection with marginal irregular enhancement, commonly developing at the bladder dome. The absence of an appreciable connection with the urinary bladder lumen allows the differentiation of an infected diverticulum from a mural abscess [50].

### 2.13. Eosinophilic Cystitis

**Figure 25:**
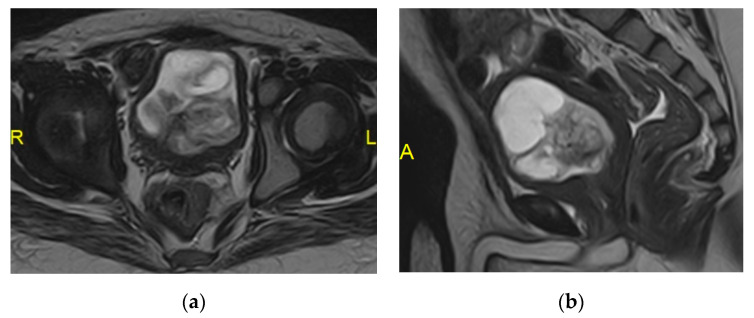
Eosinophilic cystitis. (**a**) Axial and (**b**) sagittal T2 weighted images show diffuse bladder wall thickening with an intravesical superficial mass displaying heterogeneous signal intensity. It is seen related to the posterior bladder wall with intact muscle layer; the lesion encroaches upon ureteric orifices and bladder neck. It was proved as eosinophilic cystitis by histopathology.

Eosinophilic cystitis represents a rare chronic inflammatory condition of the urinary bladder. It is characterized mainly by the infiltration of the urinary bladder wall with eosinophils associated with variable degrees of fibrosis and muscle necrosis [51]. Eosinophilic cystitis may occur in patients with peripheral eosinophilia or allergy. Hematuria, frequency, and dysuria are the most common associated symptoms [52,53]. At the radiologic assessment, a single bladder mass maybe visualized more frequently than multiple masses and may be sessile [51,53]. MRI shows mural bladder wall thickening with a mass displaying inhomogeneous variable signal intensity on T1 and T2–WI (Figure 25). The mass is usually enhanced after intravenous administration of contrast material. A cystic variant with an enhancing wall may be seen. In the fibrotic stage of the disease, the urinary bladder is small and contracted, and there may be resultant hydronephrosis [41,54].

### 2.14. Cystitis and Fistula

Colovesical fistulas occur most commonly in association with Crohn’s disease and diverticulitis. In smaller fistulae, the only clinical presentation may be chronic cystitis. Bladder cancer, iatrogenic injuries, and radiotherapy may also favor colovesical fistula formation. Frequently, larger fistulae lead to air or feces excretion via the urine [3,34,36,55].

**Figure 26:**
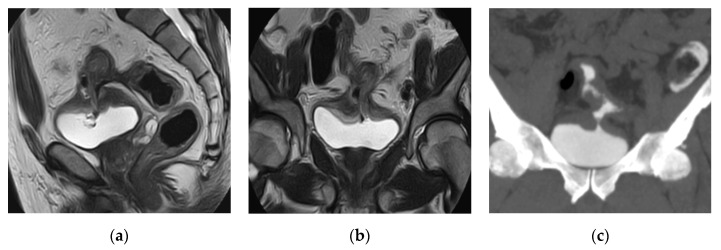
Colovesical fistula. (**a**) Sagittal and (**b**) coronal T2 weighted images show marked irregular bladder wall thickening at the bladder dome with related inflammatory soft tissue closely related to the adjacent thick-walled sigmoid colon. Intravesical signal void air locules also could be noted. (**c**) Coronal reformatted CT cystography confirms the presence of colovesical fistula.

In CT and MRI, fistula detection can be reached either directly (by filling the fistulous tract with contrast medium) or indirectly (only by showing the communication of two organs or by air detection in the fistulous tract) (Figure 26). For fistula detection, MRI is better than CT, since the fistulae can be detected at T2–WI with fat suppression sequence and after Gadolinium administration in fat-saturated T1–WI. Moreover, MRI can be used to detect a coexisting abscess via DWI with high specificity [34,36]. CT findings are usually diagnostic, in 90–100% of cases, and include endoluminal air, mural thickening, mucosal hyperemia, tethering of adjacent thick-walled bowel as well as the presence of pericolic fat stranding and dissecting inflammatory fistula tract. Multiplanar coronal and sagittal reformatted images, as well as three-dimensional reconstructions, are usually invaluable in detecting the fistula tract, particularly at the level of the bladder dome where partial volume averaging may potentially obscure the findings seen on axial images [34,41].

### 2.15. Acute Bacterial Prostatitis and Prostatic Abscess

Prostatitis has a prevalence of 9% among adult male patients. The route of contamination is usually ascending. Prostatitis can be classified into 4 categories [56] as following: I. Acute bacterial prostatitis, II. Chronic bacterial prostatitis, III. Chronic prostatitis/chronic pelvic pain syndrome (CPPS); inflammatory (IIIA) and non-inflammatory (IIIB), IV. Asymptomatic inflammatory prostatitis [42]. Fever, dysuria, frequency, and pelvic pain are common presentations. The prostate is usually enlarged and tender during the digital rectal examination. About 10% of patients with acute bacterial prostatitis progress to chronic bacterial prostatitis and a further 10% of cases progress to CPPS. Risk factors for acute bacterial prostatitis include urethral catheterization and prostate biopsy [37].

The prostatic abscess requires early diagnosis and management to avoid severe complications. Most prostatic abscesses occur secondary to lower urinary tract obstruction or hematogenous spread of infection in patients with pre-existing prostatitis. The nonspecific symptoms of prostatic abscess often make the diagnosis difficult [37,57].

**Figure 27:**
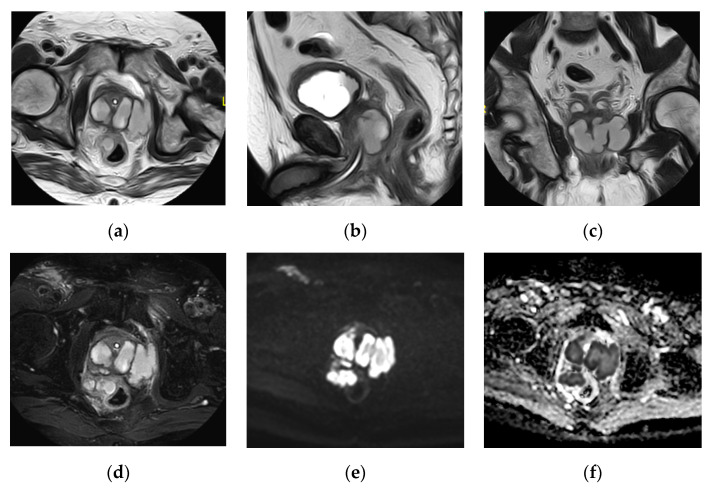
Prostatic abscess. (**a**) Axial, (**b**) sagittal, (**c**) coronal T2 weighted images and (**d**) Axial T2 weighted image with fat suppression show that the enlarged prostate is a seat of a multilocular cystic lesion with thick wall and thick septae, displaying fluid signal intensity, showing extraprostatic extension with periprostatic fat stranding and inflammatory reaction, it is seen indenting the right lateral wall of the rectum, and extends to the left obturator internus muscle. The DWI (**e**) and apparent diffusion coefficient (ADC) map (**f**) show the abscess as an area of restricted diffusion; high signal intensity (SI) at DWI and low SI at ADC map.

Transrectal ultrasound (TRUS) is indicated for patients who fail initial treatment for prostatitis and particularly for suspected prostatic abscess. TRUS can also guide abscess drainage and provide samples for culture [37]. CT has no role in the diagnosis of acute prostatitis however, it may diagnose an abscess or extraprostatic extension. Although MRI can detect the diffuse asymmetric prostate enlargement, it is usually indicated for suspected prostatic abscess, which appears as a cystic lesion with thick walls, septae, or heterogeneous contents. Signal intensity is usually isointense to hyperintense on T1-WI, and isointense to hypointense on T2-WI due to pus and debris. Thick-walled fluid collections due to abscess formation are well seen at contrast-enhanced MRI. Excellent soft-tissue characterization provided by MRI is useful to assess the prostatic abscess extension and extraprostatic involvement. Diffusion-weighted MRI will detect the prostatic abscess as an area of diffusion restriction that correlates with T2 weighted abnormality (Figure 27) [2].

### 2.16. Chronic Prostatitis

TRUS is usually normal in most cases of chronic prostatitis. Calcification can be easily detected by CT but it is not specific for chronic prostatitis. Overlapping features between chronic prostatitis and prostate cancer are also seen on MRI. Diffusion-weighted MRI shows restricted diffusion in both cancer and prostatitis, but using the ADC value < 1.2 × 10^−3^ mm^2^/s as a cutoff point for the diagnosis of cancer increased the DWI sensitivity and specificity [2].

### 2.17. Urethritis

Urethritis is commonly caused by a sexually transmitted infections. Urethritis from chlamydia, trachomatis, and gonorrhea is favored by low socioeconomic status. Alternatively, urethral infection results from permanent or intermittent catheterization or from urologic instrumentation. Symptoms include dysuria, mucopurulent discharge, and urethral pruritus [50,58].

**Figure 28:**
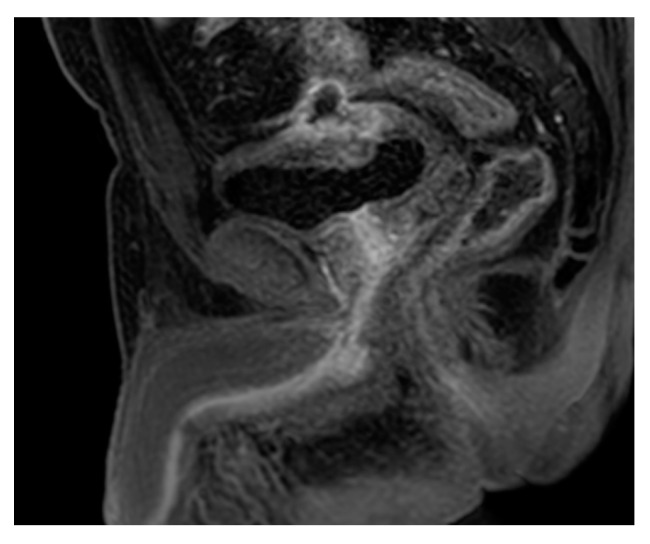
Cystitis and urethritis. Sagittal post-contrast T1 weighted image shows diffuse thick enhancing urethra suggesting urethritis. There is also cystitis complicated by an exophytic bladder wall abscess showing a thick enhancing wall.

Acute urethritis is often diagnosed on the background of clinical and laboratory findings however, imaging may be required to exclude associated complications such as a periurethral abscess. At MRI, acute urethritis appears as diffuse thickening of the urethra as well as periurethral tissues, with intermediate to high signal intensity on T2-WI and intense contrast enhancement (Figure 28). The use of MRI can effectively visualize different abnormalities related to the urethra such as perineal and periurethral abscesses [50,59].

A periurethral abscess is a life-threatening condition of the male urethra which may result from gonococcal infection, urethral stricture, or urethral catheterization. CT and MRI can demonstrate the presence and extension of a periurethral abscess and can assess the associated complications such as urethroperineal fistulas, Fournier gangrene, and fasciitis. Urethroperineal fistulas are usually the consequence of a periurethral abscess. The initial cavity of the abscess contracts due to fibrosis, leaving only the narrow fistulous tract extending from the urethra to the perineum. Urethroperineal fistulas are usually the result of urinary tuberculosis and schistosomiasis infections [60].

The characteristic cross-sectional imaging features of different types of UTIs affecting the upper and lower urinary tracts are summarized in Table 1.

## 3. Conclusions

This is an excellent review of the CT and MRI in urinary tract infections. It should interest most of the readers of *Medicina* including radiologists, urologists, and nephrologists. We should be familiar with the CT and MRI signs of these common and potentially severe disorders, which may require early diagnosis and appropriate management that decrease the incidences and degree of complications.

## Figures and Tables

**Table 1:** Cross-sectional imaging signs of different types of urinary tract infections.

Infectious Conditions	Cross-Sectional Imaging Signs
**Acute pyelonephritis**	Wedge shaped hypo-enhancing areas or striated nephrogram pattern. Perinephric fat stranding and thickening of Gerota’s fascia.
**Renal abscesses**	Round or geographic non-enhancing central fluid collection and enhancing rim. Perinephric fat stranding and thickening of Gerota’s fascia.
**Emphysematous UTIs**	Gas in the renal parenchyma, collecting system, bladder lumen and sometimes in the perirenal and perivesical tissue.
**Pyonephrosis**	Dilated thick-walled hyperenhancing collecting system, distended with high attenuation pus-filled fluid, fluid—fluid layering at T2WI, and thinning of the renal cortex.
**Chronic pyelonephritis**	Renal scarring, cortical atrophy, calyceal clubbing, thickening and dilatation of the calyceal system and overall renal asymmetry.
**Xanthogranulomatous Pyelonephritis**	Non-functioning enlarged kidney, obstructing stone within a non-dilated renal pelvis, expansion of the calyces, and inflammatory changes in the perinephric fat.
**Urinary tuberculosis**	Calyx stem stenosis with proximal ball-shaped hydrocalyx, cavity communicating with a deformed calyx, putty kidney, ureteric strictures and shortening with beaded appearance, thick-walled contracted bladder.
**Renal replacement lipomatosis**	Total atrophy of the renal parenchyma with complete fibrofatty replacement associated with stag horn stone. Stretched calyces without hyronephrosis.
**Urinary Bilharziasis**	Contracted, fibrotic, thick calcified bladder wall with ureteric stenosis and calcifications.
**Urinary candidiasis**	Rolled appearance when it contains air bubbles between the layers of fungal colonies. If air bubbles are not present, it appears as non-enhancing solid mass.
**Ureteritis**	Diffuse mucosal urothelial thickening, often with associated periureteric fat stranding.
**Encrusted pyelitis and cystitis**	Linear hyperdense calcifications along the thickened urothelium.
**Acute infectious cystitis**	Diffuse bladder wall thickening, especially if oedematous at T2 weighted image, urothelial hyperenhancement, perivesical fat stranding.
**Mural bladder abscess**	Intramural/exophytic non-enhancing fluid collection, irregular wall, often thick peripheral enhancement, usually affecting the upper bladder aspect.
**Prostatic abscess**	Non enhancing fluid collection with peripheral or septal enhancement and non-enhancing central fluid. Possible extraprostatic extension
**Acute urethritis**	Thickened urethra and surrounding soft tissues, high T2 weighted signal intensity, corresponding intense contrast enhancement.
